# When, why, and how do People Deviate from Physical Distancing Measures During the COVID-19 Pandemic: A Mixed-Methods Study

**DOI:** 10.5334/pb.1089

**Published:** 2021-09-02

**Authors:** Maité Van Alboom, Fleur Baert, Aline Wauters, Melanie Beeckman, Sarah Maes, Ama Kissi, Elke Veirman, Dimitri M. L. Van Ryckeghem, Annick De Paepe, Louise Poppe

**Affiliations:** 1 Ghent Health Psychology Lab, Department of Experimental-Clinical and Health Psychology, Faculty of Psychology and Educational Sciences, Ghent University, BE; 2 Physical Activity & Health Research Group, Department of Movement and Sports Sciences, Faculty of Medicine and Health Sciences, Ghent University, BE; 3 Section Experimental Health Psychology, Department of Clinical Psychological Science, Faculty of Psychology and Neuroscience, Maastricht University, Maastricht, the Netherlands, NL; 4 Department of Behavioural and Cognitive Sciences, Faculty of Humanities and Social Sciences, University of Luxembourg, Esch-sur-Alzette, Luxembourg; 5 Health Promotion Research Group, Department of Public Health and Primary Care, Faculty of Medicine and Health Sciences, Ghent University, BE

**Keywords:** COVID-19, physical distancing measures, HAPA, motivation for deviation

## Abstract

**Introduction:**

To limit the spread of COVID-19, many countries, including Belgium, have installed physical distancing measures. Yet, adherence to these newly installed behavioral measures has been described as challenging and effortful. Based on the Health Action Process Approach (HAPA) model, this study performed an in-depth evaluation of when, why, and how people deviated from the physical distancing measures.

**Methods:**

An online mixed-method study was conducted among Belgian adults (N = 2055) in the beginning of May 2020. Participants were recruited via an open call through email and social media platforms, using snowball sampling. Conditions wherein people deviated from the physical distancing measures were assessed by means of an open-ended question. HAPA determinants were assessed in a quantitative way.

**Results:**

Half of the sample reported to deviate from the measures. Further, deviation from the measures was associated with each determinant outlined by the HAPA. Findings highlight that many people deviated from the measures because of their need for social contact. The majority of the people who deviated from the measures stated that they carefully weighed the risks of their behavior.

**Conclusions:**

Need for social contact pushed people to deviate from physical distancing measures in a deliberate manner. Potential areas for future interventions aimed at promoting adherence to physical distancing measures and enhancing psychosocial well-being are discussed.

## Introduction

Worldwide, the COVID-19 pandemic has had an enormous impact on people’s daily functioning. To limit the spread of the coronavirus, which causes COVID-19, physical distancing measures were installed, i.e., people were instructed to stay at home as much as possible and to keep physical distance from people other than their household members. Adhering to these physical distancing measures has required massive and rapid behavioral change. Longstanding existing knowledge on behavior change can play a key role in informing policy makers and explaining the effects of these preventive measures taken in this new context.

Several recent studies have demonstrated that the personal determinants described in the Health Action Process Approach (HAPA; Schwarzer & Luszczynska, 2008) are associated with adherence to the physical distancing measures taken to reduce the spread of COVID-19 (e.g., Hamilton et al., 2020; Beeckman et al., 2020; Lin et al., 2020). The HAPA states that the process of behavior change consists of two distinct phases: (1) a *motivational phase* during which an intention for behavior change is developed and (2) a *volitional phase* during which this intention is translated into actual behavior change (Schwarzer & Luszczynska, 2008). Within the motivational phase, positive outcome-expectancies and self-efficacy are considered proximal determinants for developing an intention for change, while risk perception is described as a distal determinant. Within the volitional phase, action planning and coping planning are central processes that drive the translation of the intention into actual behavior. Hamilton et al. (2020) found that both motivational and volitional processes played a role in adhering to the physical distancing measures among Australian and US adults. Similarly, Beeckman et al. (2020) demonstrated that Belgian adults who adhered to the physical distancing measures reported higher scores on positive outcome-expectancies, self-efficacy, intention, action planning, and coping planning than those who did not adhere to these measures. Finally, Lin et al. (2020) found that COVID-19 preventive behaviors (including keeping physical distance) among Iranian adults were predominantly predicted by people’s self-efficacy, intention, action planning, and coping planning.

Rates of adherence to the physical distancing measures, however, only provide a general understanding of people’s response to these measures. Beeckman et al. (2020) found that adhering to the physical distancing measures during the first lockdown was highly challenging for more than 30% of their Belgian sample. Furthermore, over 30% reported that they would not be able to adhere to the physical distancing measures for as long as needed. Indeed, adherence to the physical distancing measures may take its toll on individuals’ well-being. People tend to experience a lack of freedom, loneliness, lack of routines, etc., which may lead to lower mental and social well-being (e.g., Barari et al., 2020; Park & Park, 2020). These negative consequences may, in turn, stimulate people to deviate from the physical distancing measures. Nevertheless, little is known about when, why, and how people deviate from these physical distancing measures. The inclusion of a more motivational perspective examining the reasons for deviation may aid in the identification of the individual needs that are potentially being frustrated among people who deviate from the physical distancing measures. This may offer additional insights to inform interventions targeting adherence to these measures.

The aim of the current study was threefold. First, we aimed to assess conditions wherein people deviated from the physical distancing measures that were established by the Belgian government during the beginning of May 2020 (i.e., staying at home, only seeing one person besides household members, keeping 1.5 m distance from others) (*When?*). A second aim was to examine the association between the HAPA-determinants (i.e., self-efficacy, outcome expectancies, risk perception, intention, action planning, and coping planning) and deviation from these physical distancing measures (*Why?*). Finally, we aimed to gain a deeper understanding of how people deviated from physical distancing measures (*How?*). To meet these aims, quantitative as well as qualitative data were collected using an online survey.

## Methods

### Participants

Participants were recruited via an open call through email and social media platforms (i.e., Facebook, Instagram and Twitter), using snowball sampling. Data of participants was excluded if they (1) did not reach the required age of 18 years (N = 13), (2) did not live in Belgium at the time of completing the survey (N = 19), (3) did not provide explicit informed consent (N = 26), or (4) indicated that they did not fill out the survey in an honest way (N = 5). Incomplete responses were not withheld in the final dataset. The final sample consisted of 2055 participants.

### Measurements

#### Sociodemographic Information

Participants’ age, gender, and education level were assessed to index their general demographic characteristics. To gain insight into participants’ occupational status, respondents were asked to indicate if they were still at work. If they answered this question affirmatively, participants were asked to indicate whether they: 1) were working onsite or offsite (i.e., at home), 2) were required to interact with people at work, and 3) could adhere to the 1.5 meter distance rule at work.

#### Personal Determinants (HAPA)

Personal determinants suggested by the HAPA (i.e., self-efficacy, outcome expectancies, risk perception, intention, action-planning, and coping planning) were assessed using questions adapted from an existing, content-valid questionnaire measuring determinants for adopting an active way of living (Poppe et al., 2019a, 2019b). Items were modified so that they were directly relevant for the COVID-19 pandemic (e.g., “I have confidence in my ability to adhere to the COVID-19 measures”).

Three items were used to assess *self-efficacy*: (1) “I have confidence in my ability to adhere to the COVID-19 measures,” (2) “I have confidence in my ability to adhere to the COVID-19 measures, even during difficult times’’, and (3) “I have confidence in my ability to adhere to the COVID-19 measures, even if they persist for a longer period of time.” Cronbach’s α for these items was excellent (α = 0.92; 95% CI = [0.91, 0.93]). *Outcome expectancies* were assessed using the following three items: (1) “If I adhere to the COVID-19 measures, I have a lower risk of getting infected,” (2) “If I adhere to the COVID-19 measures, less people will get infected,” and (3) “If I adhere to the COVID-19 measures, other people will value me for this.” The internal consistency of these items was acceptable (α = 0.77; 95% CI = [0.75, 0.79]). *Risk perception* was assessed with two items: (1) “I have little chance of getting infected with COVID-19” and (2) “If I get infected with COVID-19, I will recover quickly.” The internal consistency of these items was poor (α = 0.43; 95% CI = [0.38, 0.48]). Because of the poor internal consistency of these items and because people’s estimation of recovery might also be influenced by other variables, such as age and health status, we decided to only use the first item assessing risk perception in the analyses. This item was re-coded so that higher values indicated higher risk perception. *Action-planning* was assessed with the item “I know exactly what I am going to do (e.g., how, when, where,…) to adhere to the COVID-19 measures.” *Coping planning* was assessed using one item: (1) “I have considered potential solutions for possible obstacles (e.g., taking care of children, lack of social contact,…)”. *Intention* was indexed with one item: “I intend to adhere to the COVID-19 measures.” All items were rated on a five-point Likert scale (‘1 = totally disagree’, ‘2 = rather disagree’, ‘3 = neutral’, ‘4 = rather agree’, and ‘5 = totally agree’). Mean item scores were used in analyses for those determinants assessed with more than one item (i.e., self-efficacy and outcome expectancies).

#### Deviation from the physical distancing measures

Deviation from the physical distancing measures that were in effect at the time of the survey was measured by means of one open-ended question: “In which areas do you allow yourself to deviate from the measures?” A number of example answers were provided to facilitate responding (e.g., “I still go to my parents”, “I still meet other people’’, “People still visit me at home”).

### Procedure

The survey was online from May 1^st^ until May 9^th^ 2020. During that time, several measures issued by the Belgian government to prevent the spreading of COVID-19 were still in effect. These measures included basic hygiene measures (i.e., washing hands, coughing or sneezing in the elbow) and physical distancing measures (i.e., staying at home, only seeing one person besides household members, keeping 1.5 m distance from others, teleworking [unless impossible because of the nature of the work], and avoiding non-essential transportation). All these measures were in force as of March 18^th^ 2020. Physical activity in open air was allowed with one (until the 4^th^ of May) or two (from the 4^th^ of May on) other persons, if physical distance could be guaranteed. Additionally, on the 4th of May, shops selling fabrics were allowed to re-open. Starting from the 6th of May, it was allowed to meet other people, preferably outside, with a maximum of four people if these were always the same people. The survey was programmed in the LimeSurvey 2.00 platform. Participants were first provided with information about the study. Upon providing informed consent, participants were presented the survey, consisting of three parts: 1) questions assessing sociodemographic information, 2) questions assessing HAPA-based personal determinants, and finally, 3) the open-ended question assessing when and how people deviated from the measures.

No incentive was provided for completing the survey. The study was approved by the Ethical Review Committee of the Faculty of Psychology at Ghent University (2020/38b).

### Mixed-method analyses

Qualitative responses to the open-ended question assessing deviation from the measures were independently coded by two researchers (MVA and LH). First, a coding scheme was created based upon a straightforward content analysis of the responses to the question “In which areas do you allow yourself to deviate from the measures (e.g., “I still go to my parents”, “I still meet other people’’, “People still come to my home”). The emerging conceptual categories included in this coding scheme were developed by a multi-stage process, combining bottom-up and top-down processes (Saldaña, 2009). More specifically, the construction of the categories was informed by (1) information derived from the media (e.g., statements about feelings of loneliness), (2) the measures which were in effect during that time, and (3) the responses on the open-ended question themselves.

**Figure 1:**
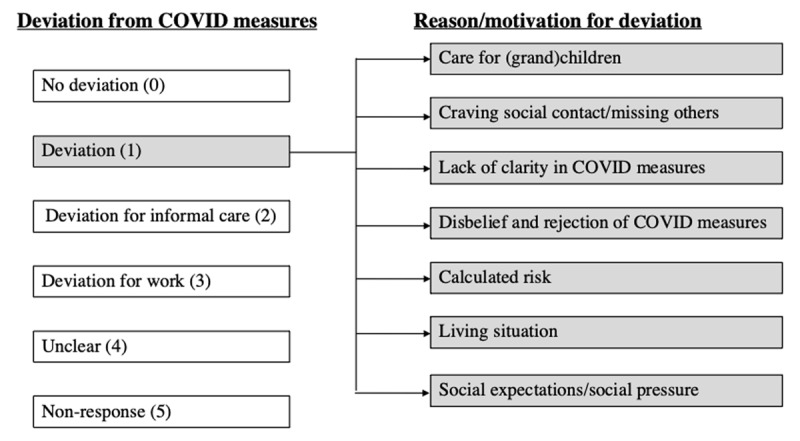
The final coding scheme.

After finishing the construction of the first version of the coding scheme, MVA and LH independently coded the first 50 qualitative responses using the scheme. Before doing so, the order of the responses was randomized. Afterwards, the correspondence between the coding of both researchers was thoroughly discussed and evaluated by the research team. Based on these discussions the codebook was fine-tuned and similar categories were merged into one category (e.g., the reasons “missing people” and “mental health” were merged into “craving for social contact/missing others”). Furthermore, illustrative examples of responses supporting each category were included in the coding scheme. The final coding scheme consisted of two levels (***Figure 1***). The first level was divided into six separate categories indicating whether the participant deviated from the measures or not, whether the deviation was related to work or informal care (which were allowed), and finally one category for unclear responses and one for empty responses. The second level was divided into seven categories and indicated the reason or motivation for the deviation. The categories within the first level were mutually exclusive, but within the second level multiple reasons could be identified for one and the same response. Three quarters of the participants did not explicitly report a reason or motivation for deviation from the measures.

Using the final coding scheme MVA and LH independently evaluated whether and how participants deviated from the measures and, if applicable, what the motivation or reason was for the deviation. MVA and LH each coded 60% of the responses of which the order was randomized. Hence, 20% of the responses were coded by both researchers. Cohen’s kappa coefficient, reflecting interrater agreement, was calculated for the first level (i.e., deviation from the measures) using the “irr” package (Gamer, 2010) in R version 4.0.3 (R Core Team, 2020). This resulted in a kappa of 0.88, reflecting a strong level of agreement (McHugh, 2012). Because the second level contained categories with only a limited number of cases (see Results) and Cohen’s kappa is strongly influenced by the prevalence of the cases (Sim & Wright, 2005), the kappa coefficient was not calculated for the second level.

Quantitative analyses were performed using R version 4.0.3 (R Core Team, 2020). Based on the six original categories for “Deviation from the COVID measures” (***Figure 1***), a binary variable was created (i.e., deviation [=deviation in the original category] vs. no deviation [=no deviation, deviation for work and deviation for informal care]). Participants who did not provide an answer to this question or who provided an unclear answer were not withheld in this part of the analyses. To investigate the association between deviation from the measures and the HAPA determinants, six regression models were fitted with the HAPA determinants as dependent variables and deviation from the measures (as obtained by the qualitative analyses) as independent variable. Age, sex, level of education, contact at work and the day on which participants filled out the questionnaire were added as covariates. Estimates with *p*-values below .05 were considered statistically significant.

## Results

### Sample Characteristics

Participants’ sociodemographic characteristics and their mean score for the HAPA determinants are presented in ***Table 1***.

**Table 1:** Sample characteristics.


CHARACTERISTICS	PARTICIPANTS (N = 2055)

Sex	

Men, N (%)	614 (30%)

Women, N (%)	1434 (70%)

Other, N (%)	7 (0.003%)

Age, mean (SD); range	44.60 (15.36); 18.00–85.00

Level of education	

Low, N (%)	799 (39%)

High, N (%)	1256 (61%)

HAPA determinants	

Self-efficacy, mean (SD); range	3.84 (0.93); 1.00–5.00

Outcome expectancies, mean (SD); range	3.92 (0.76); 1.00–5.00

Risk perception, mean (SD); range	3.25 (1.05); 1.00–5.00

Intention, mean (SD); range	4.14 (0.91); 1.00–5.00

Action planning, mean (SD); range	3.71 (0.94); 1.00–5.00

Coping planning, mean (SD); range	3.37 (1.02); 1.00–5.00


### Deviation from the Physical Distancing Measures

The frequencies and relative proportions of participants’ responses in the first level categories are presented in ***Table 2***.

**Table 2:** Frequency and proportion of participants in each category of the first level (N = 2055)**.


FIRST LEVEL CATEGORIES	N (%)

No deviation	579 (28.2)

Deviation	1020 (49.6)

Deviation for informal care	78 (3.8)

Deviation for work	39 (1.9)

Unclear	71 (3.5)

Non-response	268 (13.0)


#### No deviation

Almost one third of the participants indicated that they adhered to all physical distancing measures which were in force at the time of the survey. Some people explicitly expressed that they thought it was of crucial importance to adhere to the measures and that deviations of others gave them stress.

*“I do not deviate from the measures. This virus is not a joke. I become stressed and nervous if I see what other people are doing.”* (woman, 32 years old)

Other people stated that they adhered to the measures, but that it is getting increasingly difficult.

*“I am having difficulty not seeing my children who don’t live with me anymore and my grandchild. For the time being I am still persevering, but it is getting really difficult.”* (woman, 54 years old)

*“I do not deviate, but not being allowed to visit my family is getting more and more difficult.”* (woman, 35 years old)

#### Deviation

Half of the participants mentioned at least one deviation from the physical distancing measures. The severity of these deviations varied highly. For instance, some people only deviated from the essential transportation measure.

*“After 7 weeks I drove to my grandmother, who is still living on her own, to wish her a happy 89th birthday (no physical contact, 2 m distance, grandmother stood inside, I stood outside and I wore a self made mask). In principle it was not essential transportation, but I took all necessary precautions so that I could see my grandmother in real life for a moment.”* (woman, 41 years old)

*“Primarily non-essential transportations with the car, such as picking up a book for my education, masks from the seamstress, clay at the academy for ceramics.”* (woman, 34 years old)

*“Sometimes I visit my grandchildren and wave at them from a distance in the big garden, not indoors.”* (man, 74 years old)

Other people deviate in several areas.

*“I still pass by my mother. I sometimes invite a family member or friend to my garden, but I do keep my distance. Sometimes, I leave my face mask hanging under my chin while doing childcare at school. I let the children in childcare come closer to me than allowed and approach them also more closely if I must help them with their tasks. I let my daughter go to her boyfriend and the other way around.”* (woman, 52 years old)

*“I still see my family, I keep my distance there. Sometimes I went walking with 3 people instead of 2.”* (woman, 23 years old)

#### Deviation for informal care

Some participants reported that they had to deviate from the measures in order to take care of vulnerable people.

*“I visit my mother every week (as an informal carer).”* (woman, 40 years old)

*“I am an informal carer for my 94 year old mother who is living alone and I pass by daily, taking the social distancing and sanitary measures, and washing and disinfecting hands, into account.”* (woman, 67 years old)

#### Deviation for work

A small proportion of participants mentioned that they had to deviate from the physical distancing measures due to their work. This was often the case for people working in the social sector.

*“At work, I work with clients who have mental disabilities. Keeping 1.5 m distance is not possible.”* (woman, 54 years old)

*“I do not deviate from the measures, but in my sector we, for instance, receive insufficient personal protection equipment (bad face masks). A surrogate family home makes it very difficult to keep physical distance. For the residents it is even harder (since March 12 the lockdown has been strictly followed by my team). My colleagues and I are afraid to infect our residents because we are the only link with the outside world. We don’t get tested. Only when groups with a minimum of 2 residents and 2 colleagues (outbreak) test positive.”* (woman, 64 years old)

#### Unclear responses

Some responses were unclear in terms of whether the person deviated or not.

*“Getting the opportunity to see family.”* (woman, 37 years old)

*“Life is as before for me. Nothing has changed.”* (man, 34 years old)

### Association between Deviation from the Physical Distancing Measures and the HAPA-Determinants

**Figure 2:**
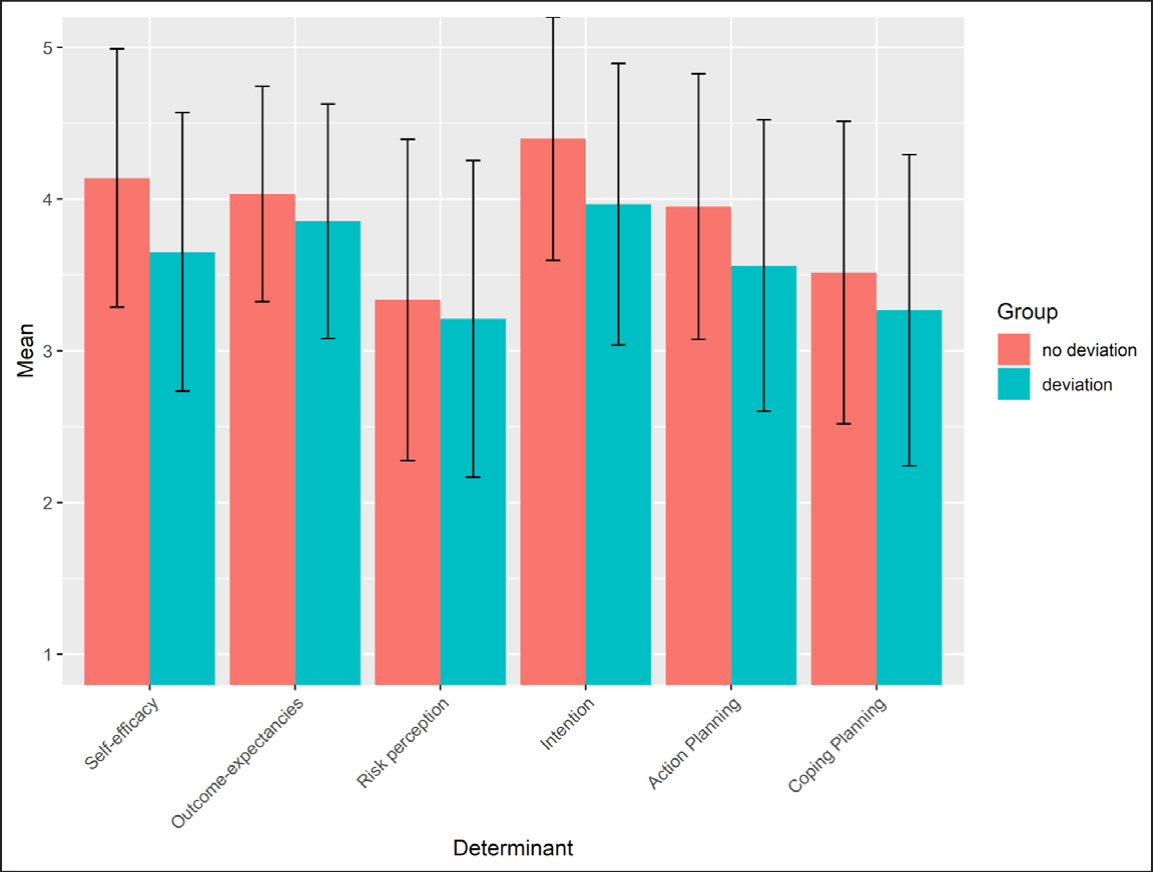
Mean and standard deviation of the score on the HAPA determinants for the group who reported to deviate from the measures and for the group who did not report to deviate from the measures.

***Figure 2*** displays the mean and standard deviation for each of the HAPA determinants for the group who reported to deviate from the measures and for the group who did not report to deviate from the measures. People who reported to deviate from the measures had significantly lower scores for self-efficacy (β = –0.42; 95% CI = [–0.51, –0.34]), positive outcome-expectancies (β = –0.17; 95% CI = [–0.24, –0.10]), risk perception (β = –0.15; 95% CI = [–0.25, –0.05]), intention (β = –0.38; 95% CI = [–0.47, –0.30]), action planning (β = –0.28; 95% CI = [–0.37, –0.20]), and coping planning (β = –0.14; 95% CI = [–0.24, –0.04]).

### Underlying Reasons for Deviation from the Physical Distancing Measures

The frequencies and relative proportions of participants’ responses in the second level categories are presented in ***Table 3***.

**Table 3:** Frequency and proportion of participants in each category of the second level (N = 504).


SECOND LEVEL CATEGORIES	N (%)

Care for (grand)children	63 (12.5)

Craving for social contact/mental well-being	89 (17.7)

Lack of clarity	10 (2.0)

Disbelief and rejection	13 (2.6)

Calculated risk	301 (59.7)

Living situation	7 (1.4)

Social expectations and pressure	21 (4.2)


#### Care for (grand)children

Many participants identified childcare as a prominent practical motivation for deviating from the measures, as at that time many daycare facilities were closed. As such, many parents struggled to efficiently reconcile their professional and household tasks and parenting duties.

*“I take the children to my in-laws, who are young and healthy people in their fifties. It’s expected of me to work from home with a baby and a toddler and I’m expected to take on the usual amount of work, which is impossible. My husband goes to work and I’m barely managing being alone with the children and working.”* (woman, 31 years old)

This struggle was magnified by circumstantial factors such as being a single parent, working in shifts or suffering from a medical condition for which the parent was on prolonged sick leave or which defined the parent as a “high-risk patient”. Also, oftentimes grandparents taking care of their grandchildren was already a part of the family routine before the pandemic outbreak, making it difficult for many families to rearrange family life around this new measure.

*“I frequently take my four-year-old son to my mother’s house, just to be able to keep it up. As a single mother writing a dissertation and unable to take my child to daycare because of the measures, I would not be able to keep this up.”* (woman, 34 years old)

*“Grandma watches the children. Taking a baby and a toddler to daycare is not safe at the moment since my husband is a high-risk patient.”* (woman, 33 years old)

Notably, many participants considered deviating from the measures in the context of child care as a last resort or a necessity due to a force majeure, often emphasizing the lack of other options. Many participants also reported on mitigating circumstances to justify their transgression, e.g., by mentioning the age and health condition of their parents or other ways in which they were extra careful.

*“I take my daughter to my parents (they are both 65 years old and in good health).”* (woman, 61 years old)

*“We take care of my brother’s children OUT OF NECESSITY, even though we shouldn’t. Their youngest is two with a weak immune system and heart failure, their eldest is six and has autism. My brother is an Intensive Care Unit nurse and his wife is a nurse at a nursing home. There is no solution (…) there is no alternative.”* (man, 45 years old)

#### Craving for social contact/mental well-being

Many participants reported deviating from the measures in an effort to reestablish social contact and intimacy with their loved ones. In this regard, the disrupted contact between grandparents and their grandchildren was a frequently reported motivation to arrange a family visit.

*“My parents have visited us. They mainly miss their grandchildren and are really struggling not being able to hug or kiss them.”* (woman, 36 years old)

Other participants reported visiting loved ones who had been isolated because of the measures (e.g., elderly (grand)parents or people living alone).

*“I will visit people who live alone or are suffering because of it or people who have just lost a loved one, from a distance whenever possible. For some people the lockdown is extremely hard!”* (woman, 28 years old)

*“I still visit my mother who lives alone and is 90 years old, mainly to keep her from feeling lonely. So I’m not adhering to the measures, but in this case my mother’s well-being is more important to me than mine.”* (woman, 46 years old)

Others emphasized their need for social contact as a way to reduce psychological distress (e.g., feelings of anxiety, depression or fatigue) brought on by the pandemic and associated measures. Some participants acknowledged that alternatives for physical social contact, such as texting and video chatting, were insufficient to meet their need for social interaction and intimacy.

*“I invited my best friend to join me in the backyard because I have not been able to sleep for the last five nights due to anxiety, I’m sorry.”* (woman, 61 years old)

*“As recommended by my therapist (I am single), I sometimes visit my best friend who is in the same age group. The lack of social contact is excruciating.”* (woman, 31 years old)

Some participants allowed themselves to start dating and initiate romantic attachments, partly motivated by the need for intimacy and daily social interaction. Couples who were not cohabitating also allowed themselves to visit each other.

*“As a single person I decided to continue dating someone. She is not my partner (yet), but it’s important for my mental health to be able to move further with this. This is obviously a risk, but for me personally, it is worth it.”* (man, 28 years old)

*“I have no difficulty adhering to the measures. The only thing I cannot stand is not being able to visit my partner (a relationship of four years).”* (woman, 21 years old)

Lastly, some participants felt compelled to reach out to loved ones outside of their family or home life in order to take a step back from difficult situations in their living situation.

*“Some situations at home are not ideal, so sometimes I feel forced to leave for a while.”* (woman, 21 years old)

#### Lack of clarity in the measures

Participants mainly reported confusion about physical distancing measures in the case of partners who were not cohabitating and emphasized that even officials (COVID hotline and police officers) could not offer more clarity.

*“It has been very unclear what is and is not allowed for people who are in a relationship but are not living together, we still have not received a clear answer to that (…) and different guidelines seem to be contradicting each other.”* (woman, 47 years old)

Some participants were confused by the perceived double standards that were inherent to the physical distancing measures, which were perceived as arbitrary or illogical.

*“I cannot see my children or grandchildren because I am taking care of my mother, but it’s okay for me to be on the bus with complete strangers? The board’s guidelines are very unclear!”* (woman, 58 years old)

#### Disbelief and rejection of the measures

Some participants questioned the authenticity of the pandemic as a whole and therefore automatically rejected physical distancing measures.

*“Because me and thousands of other people don’t believe this corona-circus whatsoever (…) It’s really starting to look like a setup to me.”* (man, 63 years old)

Some participants were not necessarily rejecting the measures or the rationale behind them, but rather conceived a personalized set of rules which they deemed useful and effective.

*“I’ll apply the useful guidelines (1,5 m distance, washing hands) and I do not wish to apply any useless guidelines. The measures could be much less strict without an increased risk.”* (man, 59 years old)

For some participants, the refusal to adhere to the physical distancing measures did not necessarily reflect a rejection of the measures themselves, but rather a resistance against the government and its authority.

*“I refuse to adhere to illegitimate decisions of an illegal government, as they are unlawfully and undemocratically appropriating the power to violate my right to self-determination, to privacy and basic human rights.”* (woman, 63 years old)

#### Calculated risk

Many participants carefully assessed the risk of contracting the virus or passing it onto others for their particular situation, taking into consideration other measures applied to compensate for the transgression. Participants frequently reported visiting others, while meeting outside, keeping their distance or wearing a face mask.

*“I still visit my grandparent who is 84 years old. Leaving her alone is not an option for us, but I do keep my distance, wear a face mask and try not to touch anything.”* (woman, 18 years old)

*“If I do deviate from the measures, I make sure I do it with people who otherwise adhere perfectly to the measures, it’s a calculated risk.”* (woman; 37 years old)

#### Living situation

Some participants’ living situation did not allow them to perfectly adhere to the measures and maintain an acceptable quality of life. Mainly participants living in larger cities, in small properties without a garden or a park nearby, expressed a great need to venture outside.

*“I meet friends in a private outdoor space, but I am adhering to the 1,5 m rule. Since I don’t have a backyard or patio, this space is very important for me to enjoy being outside. I can just sit there, in the garden and enjoy the sun.”* (woman, 47 years old)

Some participants relied on external facilities to complete their housework, such as taking laundry to a laundromat outside of their building.

*“I am staying at my own place but I don’t have the means to wash my clothes. Every two weeks, I’ll briefly meet with one of my parents and with correct social distancing to give them my laundry.”* (man, 24 years old)

#### Social expectations and social pressure

Some participants reported that their efforts to adhere to the measures as strictly as possible were hampered by family members or others not respecting the measures.

*“I don’t deviate from the measures. I don’t invite anyone. But sometimes someone stops by and it takes a lot of energy to not let them in. My partner will just let them in and I don’t feel comfortable saying anything about it.”* (woman, 29 years old)

Others noticed how loved ones or friends were much less strict in adhering to the measures and expected the same from them, which made it difficult for them to stick to the rules. They experienced a sense of social pressure to discard the measures, even though they did not feel comfortable with that.

*“I’m noticing how my family members just keep meeting and they expect me to do the same, for instance, for mothers’ day. (…) I think it’s wildly irresponsible.”* (woman, 64 years old)

## Discussion

The general aim of the current study was to examine *when, how, and why* people deviated from the physical distancing measures. A mixed-method study, underpinned by the HAPA, was performed to provide an answer to these questions.

The results of the present research can be summarized as follows: a significant proportion of our sample reported that they deviated from the measures. Further, deviation from the measures was associated with each of the HAPA-determinants and was mainly encouraged by the need for social contact or care for (grand)children. However, the majority of the people reporting to deviate from the measures highlighted that they took precautions to minimize the risk for themselves and vulnerable others.

About half of the participants reported the need to deviate from the physical distancing measures beyond the allowed deviations for work or informal care. As expected, deviations from the measures were related to lower levels of self-efficacy, positive outcome-expectancies, risk perception, intention, action planning, and coping planning. This finding is in line with other studies examining associations between the HAPA-determinants and adherence to the physical distancing measures, installed to prevent the spread of COVID-19 (Beeckman et al., 2020; Hamilton et al., 2020; Lin et al., 2020).

The majority of people who indicated to deviate from the measures argued that they carefully weighed the risks of their behavior. This instance of calculated risk-taking may imply that people knew that adhering to the measures would protect them against contamination with the coronavirus and were thus well-aware of the inherent risks of meeting people outside their household members. Indeed, the differences in average risk perception and outcome expectancies between people who reported deviation and those who did not were limited, suggesting that these are not the strongest predictors of deviating behavior. Many people described that adhering to the measures contradicted other personal goals such as “taking care of one’s mental health” or “being a good friend/family member”. This potential attitude-behavior gap might have created a sense of cognitive dissonance (Festinger, 1957) in those people, causing them to experience a discrepancy between their actual behavior and their beliefs or attitudes. Increased stress and other unpleasant feelings associated with this cognitive dissonance experience may, in turn, have motivated these people to rationalize or justify their deviation from the physical distancing measures (in order to reduce stress). This may be reflected in participants emphasizing the age or health status of the individuals with whom they have closer contacts (e.g., under 65 years old), or the ways in which they compensate for their deviation (e.g., wearing a face mask, meeting people outside). The cognitive dissonance caused by the co-existence of the physical distancing measures and the need for social proximity and its expected psychological impact have also been outlined in the study of Chakraborty et al. (2021). This potential explanation may be corroborated by the current observation of stronger differences between deviators and non-deviators in terms of self-efficacy, intention, and action planning. Because of the discrepancy between their actual behavior and their beliefs and attitudes, people might have experienced less self-efficacy to adhere to the physical distancing measures, which resulted in lower levels of intention and action planning in people deviating from the measures.

An important finding within this study was the centrality of participants’ need for closeness and social interaction with loved ones as a motivator for deviation from the measures. This finding aligns with the results of studies investigating the impact of the COVID-19 pandemic on loneliness and social well-being (Reagu et al., 2021; Banerjee & Rai, 2020). In a study by Reagu et al. (2021) participants reported the lack of contact with family members to be one of the main sources of their experienced distress. Indeed, in the context of physical distancing measures, the basic human need for closeness and belonging can be chronically frustrated in the name of collective health and safety (Hagerty et al., 1992; McLeod, 2007, Ristau, 2011). Importantly, decades of research have highlighted the potentially devastating consequences of social isolation and loneliness on individuals’ physical and mental health (House, 2001; Courtin & Knapp, 2017).

Our findings regarding the need for closeness and social interaction carry valuable insights that may inform (mental) healthcare providers, and encourage the development and implementation of social support programs catering to the social needs of those in isolation in times of physical distancing (Duan & Zhu, 2020). Further, the findings may instruct the focus and tone of public health messaging in order to effectively encourage and, importantly, support people in adhering to the measures. Barari et al. (2020) already indicated that public health messaging should not solely focus on what people should (not) do and on dissemination of knowledge, but also on how to maintain or enhance mental and social well-being while following the measures. Governments could, for instance, encourage people to attend online social activities, get fresh air everyday, or even start a new hobby.

Furthermore, governments could stimulate a more autonomous form of compliance by communicating in an autonomy-supportive and competence-fostering way (Martela et al., 2021). Consistent with the findings in our study, Martela et al. (2021) discussed that a higher feeling of autonomy stimulates long-term adherence to government rules. During the first lockdown, the sense of autonomy was lowered by very strict rules that gave little leeway in choosing how to adopt and implement the desired behaviors based on informed decisions. Our results demonstrate many attempts in creating more freedom of choice by looking for “safe” ways of deviation.

The current findings are not only of relevance for the ongoing COVID-19 pandemic, but may also provide insights into crucial determinants of human behavior in future (health) crises. Throughout the past year, knowledge regarding the current COVID-19 pandemic has substantially increased and is complemented with insights gained from numerous past epidemics. Islam, Cotler, and Jason (2020) already described lessons learned from past epidemics (i.e., the 1918 Spanish Flu, the 2009 SARS epidemic, the Ebolavirus infection), as well as from non-epidemic post-viral illnesses (e.g., tick-borne encephalitis, Epstein-Barr virus) and non-viral infections (e.g., Lyme disease). Such integration of findings could lead to an effective response to future pandemics.

The present study had several limitations. First, during the first lockdown, measures were announced at different moments and changed rapidly. In times of pandemic, these rapid changes are inevitable. Our study could not take these dynamics into account due to its cross-sectional design. Future research in this context may aim to map these dynamics. Second, as is common in self-report research assessing sensitive themes, results of this study may have been subject to initial response biases. Participants who deviated highly from the measures may have been reluctant to participate in this study and, although pseudonymization of data was guaranteed, participants may have responded in a socially desirable manner. Third, only a quarter of the participants indicated a specific reason for deviating from the measures, potentially leaving other specific reasons for deviation undetected. Fourth, highly educated women were overrepresented, which hinders the generalizability of our findings to lower educated or male populations.

In spite of these limitations, the findings are the first to shed light on conditions wherein people deviate from the physical distancing measures and reasons for doing so. Another strength is the implementation of a mixed-method design to tap into both the descriptive (quantitative) and motivational (qualitative) aspects of deviation from the physical distancing measures. Finally, our sample was large (N = 2055), thus enabling us to study the driving processes for deviation in a thorough way.

Future research is warranted to expand these findings. Ecological momentary assessment might provide additional insights into changes in personal determinants and deviation from measures on a daily basis, accounting for rapidly and frequently changing governmental measures throughout the pandemic. Further, the current study assessed deviation and motivations for doing so using a single open-ended question. Future research could qualitatively examine this in a more comprehensive way using in-depth interviews. Upcoming research could also tap into the feasibility and value of public health messaging that also focuses on enhancing the general public’s mental well-being during times of physical distancing.
